# Child marriage and its association with partner controlling behaviour against adolescent girls and young women in sub-Saharan Africa

**DOI:** 10.1186/s44263-023-00001-w

**Published:** 2023-07-31

**Authors:** Bright Opoku Ahinkorah, Richard Gyan Aboagye, Joshua Okyere, Abdul-Aziz Seidu, Eugene Budu, Sanni Yaya

**Affiliations:** 1 REMS Consult Limited, Western Region, Sekondi-Takoradi, Ghana; 2 School of Public Health, Faculty of Health, University of Technology Sydney, Sydney, Australia; 3 Department of Family and Community Health, Fred N. Binka School of Public Health, University of Health and Allied Sciences, Hohoe, Ghana; 4 Department of Population and Health, University of Cape Coast, Cape Coast, Ghana; 5 Department of Nursing, College of Health Sciences, Kwame Nkrumah University of Science and Technology, Kumasi, Ghana; 6 Centre for Gender and Advocacy, Takoradi Technical University, Takoradi, Ghana; 7 College of Public Health, Medical and Veterinary Sciences, James Cook University, Townsville, Australia; 8 Korle Bu Teaching Hospital, P. O. Box, 77, Accra, Ghana; 9 School of International Development and Global Studies, University of Ottawa, Ottawa, Canada; 10 The George Institute for Global Health, Imperial College London, London, UK

**Keywords:** Adolescent girls, Child marriage, Controlling behavour, Sub-Saharan Africa, Young women

## Abstract

**Background:**

Child marriage and partner controlling behaviours are culturally seated phenomena in sub-Saharan Africa (SSA). Child marriage refers to any legal or customary union involving a boy or girl below the age of 18. Partner controlling behaviour on the other hand refers to a situation where a sexual partner consistently tries to control their spouse’s behaviours, movements, and social contacts with other people. This study examined the association between child marriage and partner controlling behaviour among adolescent girls and young women (AGYW) in SSA.

**Methods:**

We extracted data from the most recent Demographic and Health Surveys of 26 countries in SSA. Countries whose surveys were conducted from 2010 to 2020 were included in the study. A total of 26,970 AGYW (15–24 years) were included in the study. We used a multilevel mixed-effect binary logistic regression analysis to examine the association between child marriage and partner controlling behaviour.

**Results:**

The average prevalence of child marriage was 55.40% (95% CI: 48.83–61.97). This proportion ranged from 19.62% (95% CI: 16.71–22.53) in South Africa to 85.10% (95% CI: 83.14–87.06) in Chad. The proportion of AGYW who had experienced partner controlling behaviour was 68.36% (95% CI: 64.40–72.33), and this ranged from 38.40% (95% CI: 35.55–41.25) in Burundi to 88.18% (95% CI: 83.80–92.56) in Gabon. AGYW who married as child brides were more likely [aOR = 1.31; 95% CI = 1.21, 1.43] to experience partner controlling behaviour compared to those who did not marry as child brides. AGYW in Western [aOR = 1.51; 95% CI = 1.33, 1.71] and Eastern [aOR = 1.31; 95% CI = 1.13, 1.50] part of SSA were more likely to experience partner controlling behaviour compared to those in Central Africa.

**Conclusions:**

Our study has shown that there is a significant association between child marriage and the likelihood of experiencing partner controlling behaviour in SSA. Effective policies and interventions are, therefore, needed to prevent child marriage and raise AGYW’s awareness of its implication on victims of partner controlling behaviours.

**Supplementary Information:**

The online version contains supplementary material available at 10.1186/s44263-023-00001-w.

## Background

Violence against adolescent girls and young women (AGYW) is considered a total violation of human rights and a serious public health concern [1]. This can manifest in various forms; however, spousal violence is the commonest form of violence perpetuated against AGYW [2]. It is noteworthy that spousal violence can be physical, controlling behaviours, psychological, or sexual; yet, existing empirical research have focused mainly on physical and sexual violence against AGYW [3]. Nevertheless, the issue of partner controlling behaviour is gradually gaining scholarly attention [1, 4]. In the context of this study, partner controlling behaviour refers to a situation where a sexual partner consistently tries to control their spouse’s behaviours, movements, and social contacts with other people [5].

The rising scholarly interest in the issue of partner controlling behaviour is grounded in the point that men who exhibit controlling behaviours toward their spouses are more prone to commit acts of physical, sexual, and emotional abuse against them [6]. Such controlling behaviours have serious adverse effects on the physical, psychological, and reproductive health of women [7–9]. Therefore, understanding the factors associated with partner controlling behaviour is necessary to inform the development of policies and preventive interventions.

It is important to note that partner controlling behaviour is deeply rooted in patriarchal cultural norms and belief systems, particularly in resource constrained settings such as sub-Saharan Africa (SSA) [10]. That is, societies that ride on the norm that males are superior in comparison to females or that females must totally submit to males propagate gender inequality and fuel physical, psychological, and sexual abuse that tends to be at the disadvantage of AGYW [10–12]. For this reason, the discussion of the factors associated with partner controlling behaviour must take into consideration the role of key cultural factors including child marriage.

Child marriage refers to “any legal or customary union involving a boy or girl below the age of 18” [13]. In 2019, the United Nations Children's Fund (UNICEF) estimated that one in five women living across the globe was married before their 18th birthday with South Asia (285 million) and SSA (115 million girls) having the highest prevalence of child brides worldwide [14]. This high prevalence of child marriage in SSA underscores its position as a public health concern. Child marriage has several implications and adverse effects which may include the perpetuation of intergenerational poverty as many child brides drop out of school and miss the opportunity of achieving better socio-economic status later in life [15, 16]. The socio-economic effect of child marriage may negatively affect the capacity of AGYW to be assertive and autonomous in their decision-making, and this can exacerbate the risk of partner controlling behaviours. Moreover, just like partner controlling behaviours and other forms of intimate partner violence, child marriage thrives in cultural norms and belief system that views boys as superior to girls and thus socially positions girls as inferior and inevitably obedient [13, 17]. This makes child marriage intrinsically related to partner controlling behaviours against AGYW.

Notwithstanding the shared characteristics of child marriage and partner controlling behaviours as a culturally seated phenomenon, there are no empirical studies within the sub-Saharan African region that explores the associative effect of child marriage on partner controlling behaviours. The few studies that have been conducted have focused on investigating spousal violence and its association with sociodemographic factors and husbands’ controlling behaviour [1] or changes in the lifetime prevalence of partner controlling behaviours [4]. Nevertheless, there is one study conducted in Pakistan that explored the associative effect of child marriage on partner controlling behaviours [6]. Nasrullah et al.’s study found a significant association between child marriage with partner controlling behaviours [6]. Given that child marriage and violence are both culturally rooted, it is difficult to generalize Nasrullah et al.’s study to the sub-Saharan African context. This knowledge gap in SSA presents a significant problem that warrants evidence-based research to understand the nuances with respect to child marriage and partner controlling behaviours. In this study, we hypothesized that there is an association between child marriage and partner controlling behaviour in SSA.

## Methods

### Data source and study design

Data for the study were pooled from the Demographic and Health Surveys (DHSs). We included 26 countries in SSA whose recent DHSs were conducted from 2010 to 2020. Also, these countries had data on all the variables of interest included in the study. The datasets used can be accessed at https://dhsprogram.com/data/available-datasets.cfm. According to Corsi et al. [18], DHS employed a cross-sectional design, relying on pretested structured questionnaires to collect data from the respondents: men, women, and children. DHS selected respondents using a two-stage cluster sampling technique. A predetermined number of enumeration areas (EAs) were first selected using a probability proportional to the size of the list of EAs defined in the recent population census for a particular country. A listing technique was used in the designated EAs to guarantee that each residence or household was covered. Second, households within the chosen EAs were selected using equal probability systematic sampling. The survey only included respondents whose households matched the criteria for participation. Detailed information on the sampling procedure has been highlighted in the literature [18, 19]. We included a sample of 26,970 AGYW who had observations on all the variables used in the study (Table 1). This study followed the Strengthening the Reporting of Observational Studies in Epidemiology (STROBE) guidelines [20].

**Table 1:** Sample distribution per country based on DHS data for SSA

Country	Survey year	Weighted frequency	Weighted percentage
**Central Africa**			
1. Angola	2015–2016	1179	4.4
2. Burundi	2016–2017	1104	4.1
3. Cameroon	2018	1034	3.8
4. Congo DR	2013–2014	1190	4.4
5. Rwanda	2019–2020	948	3.5
6. Chad	2014–2015	1268	4.7
7. Gabon	2012	498	1.8
**Eastern Africa**			
8. Ethiopia	2016	968	3.6
9. Kenya	2014	2025	7.5
10. Comoros	2012	364	1.4
11. Tanzania	2015–2016	966	3.6
12. Uganda	2016	1266	4.7
**Southern Africa**			
13. Malawi	2015–2016	1694	6.3
14. Namibia	2013	525	1.9
15. South Africa	2016	716	2.7
16. Zambia	2018	874	3.2
17. Zimbabwe	2015	728	2.7
**Western Africa**			
18. Burkina Faso	2010	1188	4.4
19. Benin	2017–2018	1164	4.3
20. Cote d’Ivoire	2011–2012	657	2.4
21. Gambia	2019–2020	786	2.9
22. Liberia	2019–2020	504	1.9
23. Mali	2018	750	2.8
24. Nigeria	2018	2873	10.7
25. Sierra Leone	2019	1056	3.9
26. Togo	2013–2014	645	2.4
**All countries**	**2010**–**2020**	**26,970**	**100.0**

### Sample size and study population

Our study included AGYW aged 15–24 years. In the DHS, data were collected from women of reproductive age (15–49 years) with varying marital statuses (never married, married, cohabiting, widowed, divorced, and separated). Also, partner controlling behaviour was measured for women currently in sexual relationships, and this excluded those who were widowed, divorced, separated, and not married. Hence, we included women with married and cohabiting statuses in our study. Regarding the age restriction, we restricted our sample to those aged 15–24 years to ensure the inclusion of population parameters reflecting child marriage. Several studies have utilized the same categorization of age group to examine child marriage and its influence on several health and social issues [6, 21, 22].

## Variables

### Outcome variable

Partner controlling behaviour was the outcome variable in the study. It measures the extent to which husbands/partners exercise control over their wives using the questions: is the husband jealous if his wife talks with other men? Does he accuse his wife of being unfaithful? Does he refuse to permit his wife to meet her female friends? Does he try to limit his wife’s contact with her family? And does he insist on knowing where his wife is? The response options per question were “no,” “yes,” and “don’t know.” The response options “no” and “don’t know” were recoded as “no,” while those who answered “yes” were maintained. Women who answered at least one “yes” to one of the five questions were classified as “experienced partner controlling behaviour” and this was coded as “1 = yes”. Those who replied “no” to all five questions were classified as “not experienced partner controlling behaviour” and were given the code “0 = no” [1, 23]. The distribution of the five variables used to assess partner controlling behaviour has been provided in a supplementary file (Additional file 1: Table S1).

### Key explanatory variable

Child marriage was the key explanatory variable. It was defined as marriage before 18 years of age [6]. Hence, the AGYW who married before 18 years were coded as “1 = yes [experienced child marriage]” and “0 = no [no child marriage]” for those who married when aged 18 years and above. This categorization was informed by literature that utilized the DHS dataset [24].

### Covariates

Thirteen covariates were included in the study. These covariates were selected based on the review of pertinent literature [1, 6, 23] as well as their availability in the DHS dataset. We grouped the variables into the individual level and household/contextual level. Age of the AGYW (15–19, 20–24), level of of the women and their partners (no education, primary, secondary or higher), marital status (married, cohabiting), current working status (not working, working), parity (no birth, one birth, two births, three or more births), difference in age between the woman and the partner (wife is older or same, 1–5 years older, 6–10 years older, more than 10 years older), exposure to watching television (no, yes), exposure to reading newspaper or magazine (no, yes), and exposure to listing to radio (no, yes) were the individual-level variables. The household/contextual-level variables consisted of household wealth index (poorest, poorer, middle, richer, richest), place of residence (urban, rural), and geographical subregion (East Africa, West Africa, Central Africa, Southern Africa).

### Statistical analyses

Statistical analyses were performed using Stata software version 17.0. We used forest plots to present the results of the prevalence of child marriage and partner controlling behaviour. We used cross-tabulation to determine the distribution of partner controlling behaviour across child marriage and the covariates. We employed a binary logistic regression to select significant variables for a multilevel analysis. Five multilevel binary regression models were used to examined the association between partner controlling behaviour, controlling for the individual and contextual level variables. Model O was the empty model, and it shows the variance in partner controlling behaviour attributed to the primary sampling unit (PSU) with no key explanatory variable or covariates.

We placed child marriage alone in model I. Model II contained child marriage and the individual-level covariates. We included child marriage and the contextual level covariates in model III. Model IV contained partner controlling behaviour, child marriage and the covariates. We presented the results in fixed and random effects models. The fixed-effect results showed the association between child marriage and partner controlling while controlling for the covariates. We presented the results in the fixed-effect model using crude odds ratio (cOR) and adjusted odds ratio (aOR) with their respective 95% confidence intervals (CIs). The first category in each of the variables was chosen as the reference category and assigned a value one (1.00). In the last model, random effect measured the variation in partner controlling behaviour on the PSU measured by intracluster correlation (ICC). Akaike information criterion (AIC) was used to assess the fitness and comparisons of the five models. The multilevel analysis was created in Stata using the “melogit” function. To account for disproportionate sampling, non-response, and the clustered structure of DHS data, all analyses were weighted. According to Hatt and Waters [25], pooled data can reveal broader results that are “often obscured by the noise of individual data sets”. To address this, additional adjustment to pooled data is essential to account for the variability in the number of individuals sampled in each country. At the country level, the standard weight variable for the domestic violence module (d005) was first de-normalized as follows: d005 × (total female population aged 15–49 in the country)/(total number of AGYW aged 15–24 who responded to the domestic violence module questions) and then re-normalized so that in the pooled sample the average is 1. This was important because according to the DHS sampling and household listing manual, the normalized weight is not valid for pooled data, even for data pooled for women and men in the same survey, because the normalization factor is country and sex specific [19]. Finally, all 26 countries' data were appended together into one file. At this level, we applied another weighting factor: 1/(A*nc/nt), where A is the number of countries asked a particular question, nc is the number of respondents for the country c, and nt is the sample size for the pooled data [26].

## Results

### Prevalence of child marriage and partner controlling behaviour

**Fig. 1:**
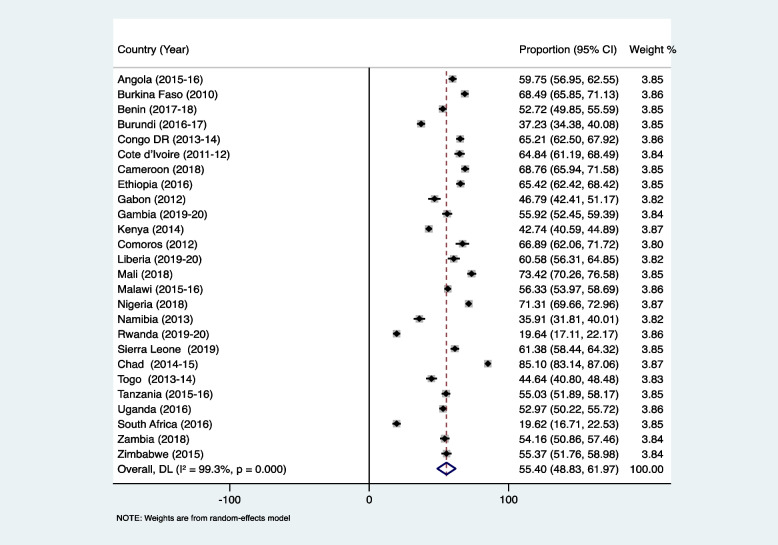
Proportion of adolescent girls and young women in sub-Saharan Africa who had experienced child marriage

**Fig. 2:**
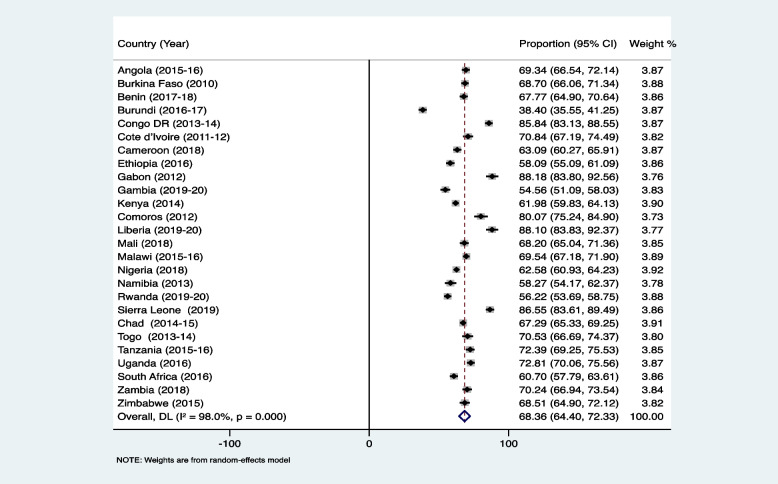
Proportion of adolescent girls and young women in sub-Saharan Africa who had experienced partner controlling behaviour

Figure 1 shows the proportion of AGYW who had experienced child marriage in SSA. The average prevalence of child marriage was 55.40% (95% CI: 48.83–61.97). This proportion ranged from 19.62% (95% CI: 16.71–22.53) in South Africa to 85.10% (95% CI: 83.14–87.06) in Chad (Fig. 1). The proportion of AGYW who had experienced partner controlling behaviour was 68.36% (95% CI: 64.40–72.33), and this ranged from 38.40% (95% CI: 35.55–41.25) in Burundi to 88.18% (95% CI: 83.80–92.56) in Gabon (Fig. 2).

### Relationship between child marriage and partner controlling behaviour

Table 2 shows the bivariable results of partner controlling behaviour across the explanatory variables. The results showed a 68.3% prevalence of partner controlling behaviour among AGYW who experienced child marriage. At the crude regression level, it was found that those who married before age 18 [aOR = 1.12; 95% CI = 1.03, 1.22] were more likely to experience partner controlling behaviour compared to those who married at age 18 and above.

**Table 2:** Bivariable results of partner controlling behaviour across the explanatory variables

Variable	Weighted	Partner controlling behaviour		
	***N* (%)**	**No (%)**	**Yes (%)**	**cOR [95% CI]**
**Child marriage**				
No (married 18 and above)	11,631 (43.1)	34.2	65.8	1.00
Yes (married below 18 years old)	15,339 (56.9)	31.7	68.3	1.12** [1.03, 1.22]
**Women’s age (years)**				
20–24	20,202 (74.9)	33.0	67.0	1.00
15–19	6768 (25.1)	32.2	67.8	1.03 [0.95, 1.13]
**Women’s educational level**				
No education	7618 (28.3)	36.4	63.6	1.00
Primary	9927 (36.8)	32.6	67.4	1.18** [1.07, 1.29]
Secondary or higher	9425 (34.9)	30.1	69.9	1.33*** [1.19, 1.48]
**Marital status**				
Married	19,456 (72.1)	34.3	65.7	1.00
Cohabiting	7514 (27.9)	29.0	71.0	1.28*** [1.15, 1.42]
**Current working status**				
Not working	12,150 (45.1)	33.7	66.3	1.00
Working	14,820 (54.9)	32.1	67.9	1.07 [0.99, 1.17]
**Parity**				
Zero birth	4852 (18.0)	33.6	66.4	1.00
One birth	10,777 (40.0)	33.2	66.8	1.02 [0.91, 1.15]
Two births	7298 (27.0)	32.3	67.7	1.06 [0.94, 1.20]
Three or more births	4043 (15.0)	31.7	68.3	1.09 [0.95, 1.25]
**Partner’s educational level**				
No education	6755 (25.1)	37.3	62.7	1.00
Primary	8259 (30.6)	34.2	65.8	1.14* [1.03, 1.26]
Secondary or higher	11,956 (44.3)	29.3	70.7	1.44*** [1.30, 1.59]
**Difference in age between the women and their partners**				
Wife older or same with partner	802 (3.0)	34.1	65.9	1.00
1–5 years older than wife	10,552 (39.1)	32.4	67.6	1.08 [0.82, 1.43]
6–10 years older than wife	9230 (34.2)	32.8	67.2	1.06 [0.80. 1.40]
More than 10 years than wife	6386 (23.7)	33.3	66.7	1.04 [0.78, 1.38]
**Exposed to watching television**				
No	17,011 (63.1)	34.2	65.8	1.00
Yes	9959 (36.9)	30.4	69.6	1.19**** [1.08, 1.31]
**Exposed to listening to radio**				
No	12,434 (46.1)	34.1	65.9	1.00
Yes	14,536 (53.9)	31.7	68.3	1.11* [1.03, 1.21]
**Exposed to reading newspaper or magazine**				
No	22,396 (83.0)	33.4	66.6	1.00
Yes	4574 (17.0)	30.1	69.9	1.16* [1.02, 1.32]
**Wealth index**				
Poorest	6135 (22.7)	33.9	66.1	1.00
Poorer	6407 (23.8)	34.3	65.7	0.98 [0.88, 1.10]
Middle	5567 (20.6)	31.5	68.5	1.12 [0.99, 1.26]
Richer	5003 (18.6)	31.7	68.3	1.11 [0.97, 1.27]
Richest	3858 (14.3)	31.8	68.2	1.10 [0.65, 1.28]
**Place of residence**				
Urban	8836 (32.8)	29.1	70.9	1.00
Rural	18,134 (67.2)	34.6	65.4	0.78*** [0.70, 0.86]
**Subregion**				
Central Africa	7222 (26.8)	34.3	65.7	1.00
Eastern Africa	5588 (20.7)	33.3	66.7	1.05 [0.92, 1.19]
Southern Africa	4537 (16.8)	33.2	66.8	1.05 [0.91, 1.22]
Western Africa	9623 (35.7)	31.2	68.8	1.15**** [1.03, 1.29]

*cO*R, crude odds ratio; *CI*, confidence interval

^*^p < 0.05, **p < 0.01, ***p < 0.001; 1.00 = reference category

### Mixed-effect analysis of the association between partner controlling behaviour and child marriage in SSA

Table 3 shows the results of the association between partner controlling behaviour and child marriage in SSA. In the complete adjusted model (model IV), it was found that those who married as child brides were more likely [aOR = 1.31; 95% CI = 1.21, 1.43] to experience partner controlling behaviour compared to those who did not marry as child brides. With the covariates, it was found that cohabiting AGYW [aOR = 1.27; 95% CI = 1.13, 1.43] were more likely to experience partner controlling behaviours compared to those who were married. Those who had secondary or higher levels of education [aOR = 1.23; 95% CI = 1.07, 1.41] were more likely to experience partner controlling behaviour. Similarly, those whose partners had primary [aOR = 1.23; 95% CI = 1.08, 1.39] and secondary or higher levels of education [aOR = 1.53; 95% CI 1.34, 1.75] were more likely to experience partner controlling behaviour compared to those with no formal education. AGYW in Western Africa [aOR = 1.51; 95% CI = 1.33, 1.71] Eastern Africa [aOR = 1.31; 95% CI = 1.13, 1.50] were more likely to experience partner controlling behaviour relative to those in Central Africa. The study also showed that those in rural areas were less likely to experience partner controlling behaviour [aOR = 0.79; 95% CI = 0.69, 0.90] compared to those in urban areas.

**Table 3:** Mixed-effect analysis of the association between partner controlling behaviour and child marriage in SSA

Variables	Model O	Model I aOR [95% CI]	Model II aOR [95% CI]	Model III aOR [95% CI]	Model IV aOR [95% CI]
**Fixed effect results**					Control
Child marriage					
No (married 18 and above)		1.00	1.00	1.00	1.00
Yes (married below 18 years old)		1.14^**^ [1.05, 1.24]	1.31^***^ [1.21, 1.43]	1.19^***^ [1.10, 1.29]	1.31^***^ [1.21, 1.43]
**Marital status**					
Married			1.00		1.00
Cohabiting			1.19^**^ [1.07, 1.34]		1.27^***^ [1.13, 1.43]
**Women’s educational level**					
No education			1.00		1.00
Primary			1.07 [0.95, 1.19]		1.13^*^ [1.01, 1.27]
Secondary or higher			1.20^**^ [1.05, 1.38]		1.23^**^ [1.07, 1.41]
**Partner’s educational level**					
No education			1.00		1.00
Primary			1.13^*^ [1.00, 1.27]		1.23^**^ [1.08, 1.39]
Secondary or higher			1.47^***^ [1.29, 1.67]		1.53^***^ [1.34, 1.75]
**Exposed to reading newspaper or magazine**					
No			1.00		1.00
Yes			0.95 [0.82, 1.11]		0.98 [0.84, 1.14]
**Exposed to listening to radio**					
No			1.00		1.00
Yes			1.09 [0.99, 1.19]		1.04 [0.95, 1.14]
**Exposed to watching television**					
No			1.00		1.00
Yes			1.15^*^ [1.03, 1.27]		1.02 [0.92, 1.14]
**Place of residence**					
Urban				1.00	1.00
Rural				0.66^***^ [0.59, 0.74]	0.79^***^ [0.69, 0.90]
**Subregion**					
Central Africa				1.00	1.00
Eastern Africa				1.28^***^ [1.12, 1.47]	1.31^***^ [1.13, 1.50]
Southern Africa				1.18^*^ [1.02, 1.37]	1.13 [0.96, 1.32]
Western Africa				1.27^***^ [1.12, 1.43]	1.51^***^ [1.33, 1.71]
***Random effect model***					
PSU variance (95% CI)	7.69 [6.44, 9.18]	7.70 [6.45, 9.20]	7.88 [6.60, 9.40]	7.82 [6.55, 9.32]	7.98 [6.69, 9.52]
ICC	0.70	0.70	0.71	0.704	0.708
Wald chi-square	Reference	9.39 (0.002)	152.68 (< 0.001)	76.05 (< 0.001)	211.28 (< 0.001)
**Model fitness**					
Log-likelihood	− 193,350.05	− 193,228.49	− 191,208.95	− 191,962.66	− 190,288.22
AIC	386,704.1	386,463.0	382,439.9	383,938	380,606.4
Total sample	26,970	26,970	26,970	26,970	26,970
Number of clusters	1306	1306	1306	1306	1306

*aOR*, adjusted odds ratio; *CI*, confidence interval

*PSU*, primary sampling unit; *ICC*, intra-class correlation; *AIC*, Akaike information criterion

^*^p < 0.05, **p < 0.01, ***p < 0.001; 1.00 = reference category

## Discussion

To facilitate sub-Saharan African countries’ achievement of the Sustainable Development Goal (SDG) target 5.3 (i.e. to end all harmful practices such as child marriage by 2030), there is a need for evidence-based research to understand the adverse effects that this practice has on AGYW to nudge behavioural change. The present study examined the association between child marriage and partner controlling behaviour in SSA. Our study showed that the pooled prevalence of child marriage and partner controlling behaviour were 55.40% and 68.36%, respectively. Also, AGYW married as child brides, those who were educated, those cohabiting, and those whose partners were educated were more likely to experience partner controlling behaviour. However, AGYW residing in rural areas were less likely to experience partner controlling behaviour.

More than half (68.36%) of AGYW in SSA had ever experienced partner controlling behaviour. The observed prevalence of partner controlling behaviour is similar to what has been reported in Nigeria (63%) [27]. However, the prevalence is higher than what was in Myanmar (30.2%) [1] and New Zealand (8.8%) [4]. This high prevalence of partner controlling behaviour in SSA highlights the endemic nature of this practice within the cultural context of the region. Also, our observed prevalence of child marriage in SSA is similar to that of Yaya et al. [28] which reported a prevalence of 54%. Thus, indicating that many AGYW in SSA are becoming brides before age 18. The high prevalence of child marriage in SSA could be attributed to early pregnancy—to avoid shame, parents may force their children to marry the person responsible for the pregnancy [29]. Other plausible explanations for the high prevalence of child marriage in SSA could include poor wealth status, harmful practices like female genital mutilation, exposure to mass media, and community literacy [30].

The hypothesis that there is a significant association between child marriage and partner controlling behaviour was substantiated by the findings of this study. Child brides were more likely to experience partner controlling behaviour compared to AGYW who did not marry as child brides. This result is consistent with the findings of a related study conducted in Pakistan that found that child brides were more likely to experience partner controlling behaviour compared to those who did not marry before age 18 [6]. Child marriage may exacerbate intergenerational poverty as many child brides drop out of school and miss the opportunity of achieving better socioeconomic status, hence significantly reducing their capacity to be assertive and autonomous in their decision-making [15, 16]. This pathway makes it easy for men to exert controlling behaviours in a bid to make AGYW subservient. Also, our finding highlights the significance of traditional elements that have been linked to violence perpetrated against women, including patriarchal nonegalitarian expectations for those married as minors and men’s conventional attitude toward women [6]. While our findings mostly concur with the hypothesis that child marriage is significantly associated with partner controlling behaviour, there was significant heterogeneity between countries with those in the Western and Eastern parts of the sub-region being more likely to experience partner controlling behaviour. Thus, emphasizing the integral role of the diverse African tradition in the perpetuation of both child marriage and partner controlling behaviour.

Additionally, educated AGYW were more likely to experience partner controlling behaviour relative to those uneducated. Similarly, AGYW whose partners were educated were more likely to experience partner controlling behaviour. Although our results seem unexpected; issues such as the exhibition of extremely stringent controlling behaviour and increased resentment from male partners towards empowered AGYW could have accounted for the observed findings in our study. Possibly, our results could have been influenced by a potential response bias whereby the educated AGYW were more likely to identify and respond to issues surrounding partner controlling behaviour relative to those uneducated. We, therefore, propose that further studies should be done to ascertain the factors contributing to this association found in our study.

### Public health and policy implications

Our findings have critical implications for policy and practice. If left unaddressed, the high prevalence of partner controlling behaviours may derail sub-Saharan African countries from achieving target 5.3 of the SDGs. Given that child marriage emerged as a risk factor for partner controlling behaviour, it is imperative for policies that aim at reducing the incidence of partner controlling behaviour to consciously focus on addressing the practice of child marriage.

### Strengths and limitations

This study’s strength lies in the use of nationally representative data and also the use of appropriate statistical to examine the association between child marriage and partner controlling behaviour. However, because the study used cross-sectional survey data, it is difficult to establish causality in the association between child marriage and partner controlling behaviour. Additionally, given the sensitive nature of child marriage and partner controlling behaviour, there is the likelihood of social desirability bias that may result in the under-reporting of the phenomena. Also, the self-reported nature of the data may result in recall bias.

## Conclusions

There is a significant association between child marriage and the likelihood of experiencing partner controlling behaviours in SSA. Effective policies and interventions are therefore needed to prevent child marriage and raise AGYW’s awareness of its implication of becoming a victim of partner controlling behaviours.

## Supplementary Information

**Additional file 1: Table S1. **Descriptive results of five variables used to measure partner controlling behaviour.

## Data Availability

All data used in this study are publicly available from DHS at http://dhsprogram.com/data/available-datasets.cfm.
