# Supplementary material for: Child marriage and its association with partner controlling behaviour against adolescent girls and young women in sub-Saharan Africa
Source: BMC Glob Public Health. 2023 Jul 31;1:9. doi: 10.1186/s44263-023-00001-w (PMC11587766; doi:10.1186/s44263-023-00001-w)
Supplement: Supplementary file 1 — Additional file 1: Table S1. Descriptive results of five variables used to measure partner controlling behaviour. [file 44263_2023_1_MOESM1_ESM.docx]

**Table S1. Descriptive results of five variables used to measure partner controlling behaviour**

| **Variable** | **No** | **Yes** | **Don’t know** |
| --- | --- | --- | --- |
| Husband/partner jealous if respondent talks with other men | 12,196 (45.22) | 14,757 (54.72) | 17 (0.07) |
| Husband/partner accuses respondent of unfaithfulness | 21,075 (78.14) | 5,884 (21.82) | 11 (0.04) |
| Husband/partner does not permit respondent to meet female friends | 20,990 (77.82) | 5,972 (22.14) | 8 (0.03) |
| Husband/partner tries to limit respondent's contact with family | 23,239 (86.17) | 3,723 (13.80) | 8 (0.03) |
| Husband/partner insists on knowing where respondent is | 14,794 (54.85) | 12,166 (45.11) | 10 (0.04 |
